# Intratumoral and peritumoral MRI-based radiomics for predicting extrapelvic peritoneal metastasis in epithelial ovarian cancer

**DOI:** 10.1186/s13244-024-01855-w

**Published:** 2024-11-22

**Authors:** Xinyi Wang, Mingxiang Wei, Ying Chen, Jianye Jia, Yu Zhang, Yao Dai, Cai Qin, Genji Bai, Shuangqing Chen

**Affiliations:** 1 Department of Radiology, The Affiliated Suzhou Hospital of Nanjing Medical University, Suzhou Municipal Hospital, Gusu School, Nanjing Medical University, Suzhou, Jiangsu China; 2 Department of Radiology, The Affiliated Huaian No. 1 People’s Hospital of Nanjing Medical University, Huaian, Jiangsu China; 3 Department of Radiology, The Fourth Affiliated Hospital of Soochow University, Suzhou, Jiangsu China; 4 Department of Radiology, The First Affiliated Hospital of Soochow University, Suzhou, Jiangsu China; 5 Department of Radiology, Tumor Hospital Affiliated to Nantong University, Nantong, Jiangsu China

**Keywords:** Ovarian neoplasms, Neoplasm metastasis, Magnetic resonance imaging, Radiomics

## Abstract

**Objectives:**

To investigate the potential of intratumoral and peritumoral radiomics derived from T2-weighted MRI to preoperatively predict extrapelvic peritoneal metastasis (EPM) in patients with epithelial ovarian cancer (EOC).

**Methods:**

In this retrospective study, 488 patients from four centers were enrolled and divided into training (*n* = 245), internal test (*n* = 105), and external test (*n* = 138) sets. Intratumoral and peritumoral models were constructed based on radiomics features extracted from the corresponding regions. A combined intratumoral and peritumoral model was developed via a feature-level fusion. An ensemble model was created by integrating this combined model with specific independent clinical predictors. The robustness and generalizability of these models were assessed using tenfold cross-validation and both internal and external testing. Model performance was evaluated by the area under the receiver operating characteristic curve (AUC). The Shapley Additive Explanation method was employed for model interpretation.

**Results:**

The ensemble model showed superior performance across the tenfold cross-validation, with the highest mean AUC of 0.844 ± 0.063. On the internal test set, the peritumoral and ensemble models significantly outperformed the intratumoral model (AUC = 0.786 and 0.832 vs. 0.652, *p* = 0.007 and *p* < 0.001, respectively). On the external test set, the AUC of the ensemble model significantly exceeded those of the intratumoral and peritumoral models (0.843 vs. 0.750 and 0.789, *p* = 0.008 and 0.047, respectively).

**Conclusion:**

Peritumoral radiomics provide more informative insights about EPM than intratumoral radiomics. The ensemble model based on MRI has the potential to preoperatively predict EPM in EOC patients.

**Critical relevance statement:**

Integrating both intratumoral and peritumoral radiomics information based on MRI with clinical characteristics is a promising noninvasive method to predict EPM to guide preoperative clinical decision-making for EOC patients.

**Key Points:**

- Peritumoral radiomics can provide valuable information about extrapelvic peritoneal metastasis in epithelial ovarian cancer.
- The ensemble model demonstrated satisfactory performance in predicting extrapelvic peritoneal metastasis.
- Combining intratumoral and peritumoral MRI radiomics contributes to clinical decision-making in epithelial ovarian cancer.

**Graphical Abstract:**

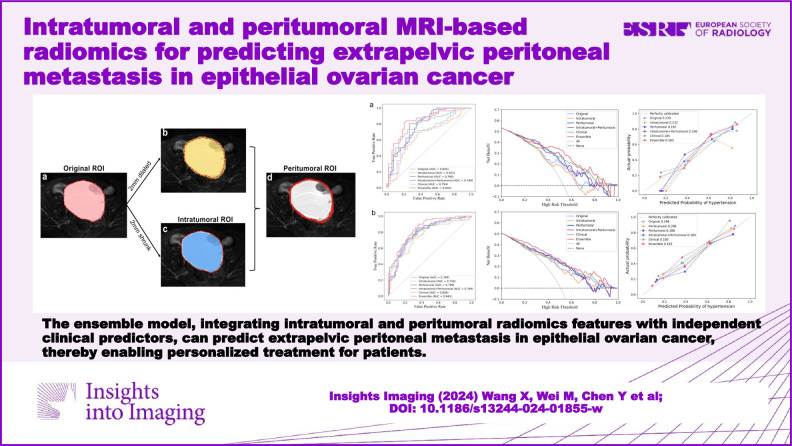

## Introduction

Ovarian cancer is the most lethal gynecological cancer, with less than a 50% 5-year survival rate [1]. It accounted for approximately 3.4% of cases and 4.7% of cancer deaths worldwide in 2020 [2]. Epithelial ovarian cancer (EOC) accounts for over 90% of all cases of ovarian malignancies. About 75% of EOC patients are diagnosed at an advanced stage of the disease, which contributes significantly to the high incidence and mortality rates [3, 4]. Extrapelvic peritoneal metastasis (EPM) refers to tumor confirmed to have spread to the peritoneum above the pelvic brim and is a distinctive characteristic of advanced-stage EOC according to the International Federation of Gynecology and Obstetrics (FIGO) [5]. Accurate preoperative assessment of the EPM status is crucial for personalized treatment and improving the prognosis of EOC patients. This assessment can influence EOC patients’ referral, the availability of fine needle aspiration biopsy, the use of neoadjuvant chemotherapy (NACT), and the selection of surgical approaches [6–9].

Recently, magnetic resonance imaging (MRI) has emerged as a potential standard imaging tool for advanced ovarian cancer, aiding in providing noninvasive preoperative assessments for patients [10]. However, the interpretation of MRI heavily relies on radiologists’ experience, thus lacking objectivity to a certain extent [11]. Inexperienced radiologists may overlook some small tumor deposits in the peritoneum, which could lead to the risk of secondary surgery or delayed treatment in patients with EOC [12, 13]. Hence, there is a need for an objective and accurate method for the auxiliary assessment of EPM.

Radiomics, which can analyze quantitative features that are invisible to the naked eye in imaging, is one of the most promising emerging methods for providing objective and accurate imaging assessment [14]. Some previous studies demonstrated that radiomics based on MRI can predict the status of peritoneal metastasis (PM) in EOC patients [15, 16]. However, these studies often fail to distinguish between intrapelvic PM and EPM and have had some limitations, such as a small sample size and a lack of external validation. Additionally, they ignore the significance of the peritumoral microenvironments in EOC. Recent studies have demonstrated that peritumoral radiomics features can provide insights into early prediction of recurrence and chemotherapy response in ovarian cancers [17, 18]. However, the utility of peritumoral radiomics features in predicting EPM in EOC patients remains unclear.

Therefore, in this multicenter retrospective study, we aimed to explore the value of intratumoral and peritumoral radiomics based on MRI for predicting EPM in patients with EOC and develop robust and interpretable models for potential clinical application.

## Materials and methods

### Patients

**Fig. 1:**
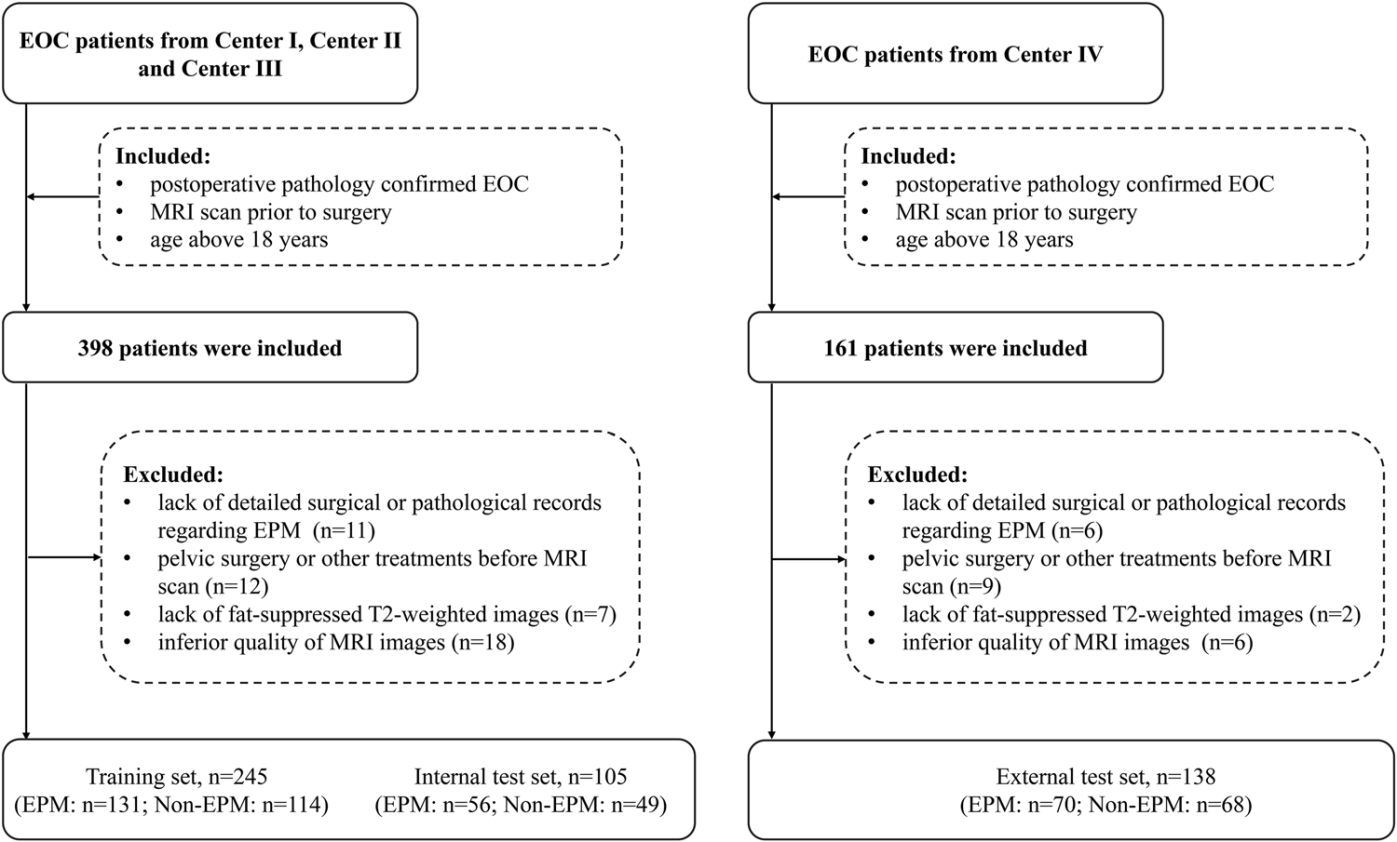
Flowchart of patient inclusion and exclusion. EOC, epithelial ovarian cancer; MRI, magnetic resonance imaging; EPM, extrapelvic peritoneal metastasis

This retrospective study was approved by the local institutional review board (K-2022-096-H01), which waived the requirement for written informed consent. Potential EOC patients for this retrospective study were consecutively enrolled from four centers (Supplementary Material Section 1) from July 2013 to July 2023. Inclusion criteria were as follows: (1) postoperative pathology confirmed EOC; (2) MRI scan performed within 2 weeks prior to surgery; (3) age above 18 years. Exclusion criteria were as follows: (1) lack of detailed surgical or pathological records regarding EPM; (2) pelvic surgery or other treatments before MRI scan; (3) incomplete MRI sequences; (4) inferior quality of MRI images, such as those having artifacts. Finally, 488 patients with EOC were included (Center I, *n* = 180; Center II, *n* = 112; Center III, *n* = 58; Center IV, *n* = 138). Patients from Centers I to III were randomly divided into a training set and an internal test set in a ratio of 7:3. Patients from Center IV constituted the external test set. The diagram of patient inclusion and exclusion is shown in Fig. 1.

Demographic and clinical characteristics, including age; FIGO stage; type; parity; menopausal status; abdominal symptoms, such as pain or distention; levels of carbohydrate antigen 125 (CA125); levels of human epididymis protein 4 (HE4); and histopathological subtype, were collected from the clinical record system.

### Evaluation of EPM

All patients underwent primary debulking surgery involving at least hysterectomy, bilateral salpingo-oophorectomy, and omentectomy. The intraoperative exploration of all peritoneal surfaces was also performed, involving the excision of all visible and resectable peritoneal implants. In addition, random biopsies were also taken from the pelvic peritoneum, both paracolic gutters and subdiaphragmatic surfaces. All obtained surgical and biopsy specimens underwent review by board-certified pathologists at the corresponding institutions. According to FIGO [5], the presence of metastasis in the peritoneum above the pelvic brim was defined as EPM (Supplementary Material Section 2).

### Image acquisition and preprocessing

**Fig. 2:**
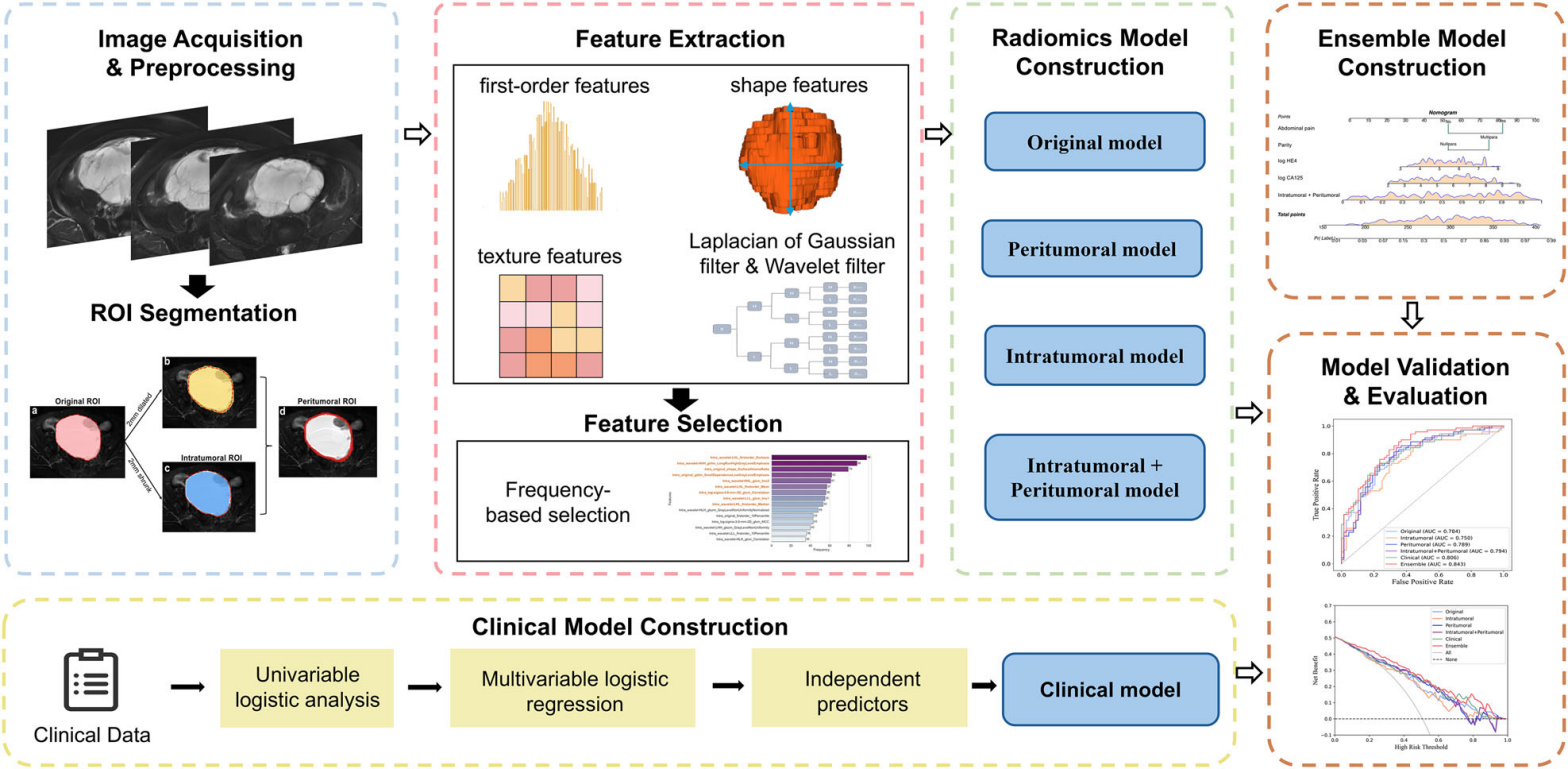
The schematic workflow of this study. ROI, region of interest

The workflow of this study is illustrated in Fig. 2. MRI images were acquired using 1.5-T or 3.0-T scanners equipped with a phased-array coil. Fat-suppressed T2-weighted (FS-T2W) images were selected for model development. Detailed FS-T2W parameters are shown in Supplementary Table S1. All images underwent N4 bias correction and were resampled to a 1 × 1 × 1 mm^3^ voxel size using B-Spline interpolation. Image intensities were then normalized to a range of 0 to 1 using minimum-maximum normalization.

### Image segmentation

**Fig. 3:**
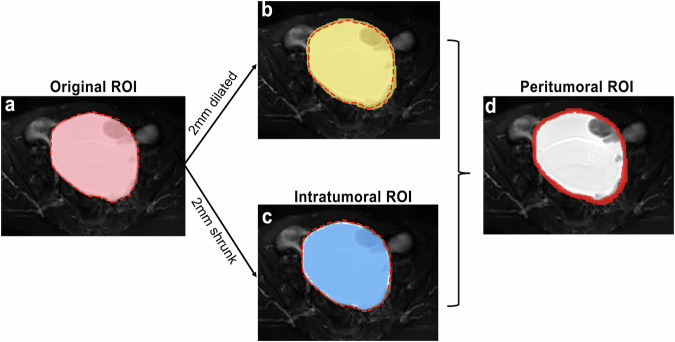
An example of the original, intratumoral, and peritumoral ROIs in a 64-year-old patient with left clear cell carcinoma. The “red dotted line” represents the tumor boundary. **a** The original ROI was the area within the tumor boundary; **b** 2 mm dilation of the tumor boundary; **c** the intratumoral ROI was derived from 2 mm shrinkage of the tumor boundary; **d** the peritumoral ROI was a 4 mm-thick ring, achieved by dilating the tumor boundary 2 mm outward and shrinking it 2 mm inward. ROI, region of interest

The original region of interest (ROI) was the area within the tumor boundary; the intratumoral ROI was the area by shrinking the original ROI inward by 2 mm; the peritumoral ROI was a 4 mm-thick ring from 2 mm dilation and shrinkage from the original ROI boundary (Fig. 3) [19, 20]. Original ROIs were manually delineated slice-by-slice on FS-T2W images using ITK-SNAP software (version 3.8.0, http://www.itksnap.org). Intratumoral and peritumoral ROIs were automatically created using SimpleITK package in Python.

Radiologist A, with 10 years of experience, segmented original ROIs for all patients. The original ROI included both solid and cystic components of the lesion. For multifocal lesions, only the largest mass was selected for delineation. Radiologist B, with 5 years of experience, segmented images from 30 randomly selected patients. After a one-month washout period, Radiologist A repeated the delineation for these patients. The intraclass correlation coefficient (ICC) was utilized to evaluate both inter- and intra-observer consistency, with an ICC > 0.80 indicating high consistency. All radiologists were blinded to patients’ histopathologic results and EPM status during segmentation.

### Feature extraction and selection

A total of 1130 radiomics features were extracted from each ROI by using the PyRadiomics package, including shape features, first-order features, second-order features, and features from image filtering. The features were then normalized using Z-score, and features with ICC < 0.80 were eliminated. To select features with high reproducibility, the training set underwent 100 random samplings, with 90% of the training data used for feature selection in each sampling. Steps for feature selection included applying the Mann–Whitney *U* test, Spearman correlation analysis, and the least absolute shrinkage and selection operator algorithm. Features that appeared with a frequency of at least 50% after 100 iterations were utilized for the final model development. Detailed information regarding feature extraction and selection is shown in Supplementary Material Section 3.

### Radiomics model development

The intratumoral, peritumoral, and original models were respectively developed based on the radiomics features from corresponding ROIs. To further investigate whether combining intratumoral and peritumoral information can enhance diagnostic efficiency, feature-level fusion was performed. This involved merging intratumoral and peritumoral radiomics features into a unified feature set, followed by feature selection and model construction. The support vector machine algorithm was employed to construct these radiomics models. During the training process for each model, the hyperparameters of the algorithm were optimized using a grid search method. The radiomics model development is shown in Supplementary Fig. S1.

### Clinical and ensemble model development

Univariable logistic regression was performed on clinical characteristics in the training set, including age, menopausal status, parity, abdominal pain, abdominal distension, and serum CA125 and HE4 levels. The serum CA125 and HE4 levels were log-transformed to normalize skewed distributions [21, 22]. Clinical characteristics with a *p* < 0.1 were subsequently included in a multivariable logistic regression to identify independent clinical predictors, which were used to construct the clinical model.

The output probability of the combined intratumoral and peritumoral radiomics model and independent clinical predictors served as inputs to the multivariable logistic regression to construct the ensemble model, visualized via a nomogram.

### Model evaluation

Each model’s robustness was assessed using tenfold cross-validation (detailed in Supplementary Fig. S2), and generalizability was evaluated using both internal and external test sets. Evaluation metrics included the area under the receiver operating characteristic (ROC) curve (AUC), accuracy, sensitivity, specificity, positive predictive value (PPV), and negative predictive value (NPV). The results of tenfold cross-validation were summarized as mean ± standard deviation. The Brier score and the calibration curve were employed to compare predicted probabilities with actual outcomes. The decision curve analysis (DCA) was used to assess the clinical net benefit of models. The SHapley Additive exPlanations (SHAP) method was utilized to explain the radiomics models by quantifying the impact of each feature on the model’s predictions.

### Statistical analysis

The statistical analysis was performed using SPSS (version 25, IBM), and models were constructed and evaluated using Python (version 3.8.5) and R (version 4.1.2). Continuous variables with a normal distribution were expressed as mean ± standard deviation, and those with a non-normal distribution were expressed as the median (interquartile range). For continuous variables, the Student’s *t*-test or Mann–Whitney *U* test was applied, depending on the distribution. For categorical variables, the chi-square test or Fisher’s exact test was used. The AUCs between models were assessed using the DeLong test. A two-tailed *p* < 0.05 was considered statistically significant.

## Results

### Clinical characteristics

A total of 488 EOC patients (mean age: 56.24 years; range: 19 to 84 years) were enrolled in this present study, including 257 EPM patients and 231 non-EPM patients. Significant differences were observed between the EPM and non-EPM groups in terms of age (*p* = 0.021), parity (*p* = 0.014), abdominal pain (*p* < 0.001), abdominal distention (*p* < 0.001), FIGO stage (*p* < 0.001), type (*p* < 0.001), log CA125 (*p* < 0.001), log HE4 (*p* < 0.001) and pathology subtype (*p* < 0.001). All patients were further subdivided into training (*n* = 245), internal test (*n* = 105), and external test (*n* = 138) sets. Detailed demographic and clinical characteristics of the patients are presented in Table 1 and Supplementary Tables S2–S4. Detailed surgical findings are presented in Supplementary Material Section 4.

**Table 1:** Demographic and clinical characteristics of all patients

	Non-EPM (*n* = 231)	EPM (*n* = 257)	*p*-value
Age			0.021*
< 55	123 (53.2%)	110 (42.8%)	
≥ 55	108 (46.8%)	147 (57.2%)	
Menopausal status			0.208
Premenopausal	37 (16.0%)	31 (12.1%)	
Postmenopausal	194 (84.0%)	226 (87.9%)	
Parity			0.014*
Nullipara	68 (29.4%)	51 (19.8%)	
Multipara	163 (70.6%)	206 (80.2%)	
Abdominal pain			< 0.001*
No	143 (61.9%)	114 (44.4%)	
Yes	88 (38.1%)	143 (55.6%)	
Abdominal distention			< 0.001*
No	156 (67.5%)	123 (47.9%)	
Yes	75 (32.5%)	134 (52.1%)	
FIGO stage			< 0.001*
I/II	205 (88.7%)	0 (0.0%)	
III/IV	26 (11.3%)	257 (100.0%)	
Type			< 0.001*
I	130 (56.3%)	35 (13.6%)	
II	101 (43.7%)	222 (86.4%)	
log CA125 (µ/mL)	4.76 ± 1.52	6.23 ± 1.38	< 0.001*
log HE4 (pmol/L)	4.80 ± 0.86	5.86 ± 0.98	< 0.001*
Pathology subtype			< 0.001*
HGSC	101 (43.7%)	219 (85.2%)	
LGSC	17 (7.4%)	10 (3.9%)	
Clear cell	44 (19.0%)	9 (3.5%)	
Endometrioid	35 (15.2%)	7 (2.7%)	
Mucinous	33 (14.3%)	7 (2.7%)	
Seromucinous	0 (0.0%)	1 (0.4%)	
Carcinosarcoma^#^	0 (0.0%)	2 (0.8%)	
Brenner	1 (0.4%)	2 (0.8%)	

Categorical variables are presented as numbers (%), and continuous variables are presented as mean ± standard deviation

*EPM* extrapelvic peritoneal metastasis, *FIGO* International Federation of Gynecology and Obstetrics, *CA125* carbohydrate antigen 125, *HE4* human epididymis protein 4, *HGSC* high-grade serous carcinoma, *LGSC* low-grade serous carcinoma

* *p* < 0.05

^#^ Carcinosarcoma is defined as epithelial origin according to the latest World Health Organization classification criteria

### Feature selection and model construction

After performing feature selection, the retained features for model development included 12 from the peritumoral ROI, 9 from the intratumoral ROI, 8 from the original ROI, and 13 from combining intratumoral and peritumoral features (Supplementary Fig. S3). These features were utilized to construct corresponding intratumoral, peritumoral, original, and combined intratumoral and peritumoral models. The log CA125 (*p* = 0.001), log HE4 (*p* = 0.005), parity (*p* = 0.033), and abdominal pain (*p* < 0.001) were independent clinical predictors (Supplementary Table S5), which were used to develop the clinical model. The ensemble model was constructed by integrating the outputs from the combined intratumoral and peritumoral model with independent clinical predictors (Supplementary Fig. S4).

### Comparison of models

The ensemble model demonstrated superior performance across the tenfold cross-validation, achieving the highest mean AUC of 0.844 ± 0.063. ROC curves across the tenfold cross-validation for each model are provided in Supplementary Fig. S5. Detailed performance for all models across cross-validation is provided in Table 2.

**Table 2:** Diagnostic performances of different models across tenfold cross-validation

Models	Accuracy	Sensitivity	Specificity	PPV	NPV	AUC
Peritumoral	0.724 ± 0.113	0.786 ± 0.134	0.665 ± 0.158	0.723 ± 0.150	0.740 ± 0.150	0.789 ± 0.099
Intratumoral	0.650 ± 0.125	0.738 ± 0.158	0.558 ± 0.126	0.652 ± 0.138	0.658 ± 0.194	0.733 ± 0.092
Original	0.669 ± 0.097	0.664 ± 0.166	0.708 ± 0.154	0.716 ± 0.166	0.656 ± 0.143	0.742 ± 0.079
Intratumoral + peritumoral	0.703 ± 0.125	0.703 ± 0.125	0.703 ± 0.125	0.703 ± 0.125	0.703 ± 0.125	0.803 ± 0.099
Clinical	0.719 ± 0.100	0.739 ± 0.128	0.696 ± 0.170	0.744 ± 0.114	0.715 ± 0.136	0.794 ± 0.081
Ensemble	0.756 ± 0.070	0.786 ± 0.135	0.717 ± 0.132	0.773 ± 0.091	0.763 ± 0.115	0.844 ± 0.063

The results of tenfold cross-validation are expressed as mean ± standard deviation

*PPV* positive predictive value, *NPV* negative predictive value, *AUC* area under the curve, *CI* confidence interval

**Fig. 4:**
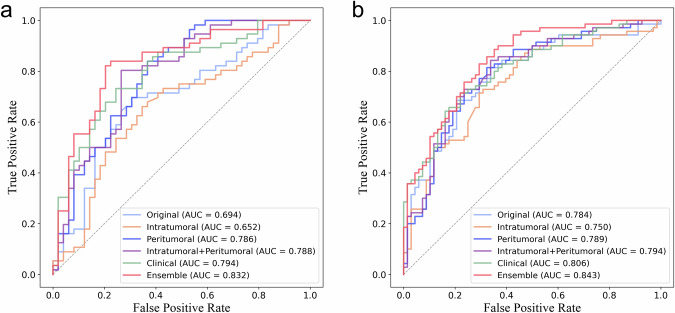
The receiver operating characteristic curves of different models for predicting extrapelvic peritoneal metastasis in epithelial ovarian cancer on the internal test set (**a**) and external test set (**b**). AUC, area under the curve

On the internal test set, the peritumoral model, combined intratumoral and peritumoral model, and ensemble model achieved AUCs of 0.786 (95% confidence interval [CI] 0.695, 0.872), 0.788 (95% CI 0.699, 0.871), and 0.832 (95% CI 0.753, 0.908), respectively, which were all significantly superior to the intratumoral model (AUC = 0.652 [95% CI 0.538, 0.749], *p* = 0.007, 0.002 and *p* < 0.001, respectively) and the original model (AUC = 0.694 [95% CI 0.591, 0.792], *p* = 0.025, 0.007 and 0.001, respectively) (Fig. 4a). On the external test set, the AUC of the ensemble model significantly exceeded those of the intratumoral and peritumoral models (0.843 [95% CI 0.783, 0.900] vs. 0.750 [95% CI 0.667, 0.831] and 0.789 [95% CI 0.712, 0.861], *p* = 0.008 and 0.047, respectively) (Fig. 4b). Delong tests between AUCs are provided in Supplementary Table S6. Detailed performance for all models on both internal and external test sets is shown in Table 3.

**Table 3:** Diagnostic performances of different models on internal and external test sets

	Models	Accuracy	Sensitivity	Specificity	PPV	NPV	AUC (95% CI)
Internal test set	Peritumoral	0.714	0.750	0.673	0.724	0.702	0.786 (0.695, 0.872)
	Intratumoral	0.657	0.679	0.633	0.679	0.633	0.652 (0.538, 0.749)
	Original	0.686	0.643	0.735	0.735	0.643	0.694 (0.591, 0.792)
	Intratumoral + peritumoral	0.771	0.804	0.735	0.776	0.766	0.788 (0.699, 0.871)
	Clinical	0.733	0.732	0.735	0.759	0.706	0.794 (0.703, 0.874)
	Ensemble	0.800	0.804	0.796	0.818	0.780	0.832 (0.753, 0.908)
External test set	Peritumoral	0.732	0.786	0.676	0.714	0.754	0.789 (0.712, 0.861)
	Intratumoral	0.688	0.657	0.721	0.708	0.671	0.750 (0.667, 0.831)
	Original	0.681	0.543	0.824	0.760	0.636	0.784 (0.701, 0.859)
	Intratumoral + peritumoral	0.732	0.771	0.691	0.720	0.746	0.794 (0.718, 0.866)
	Clinical	0.725	0.657	0.794	0.767	0.692	0.806 (0.737, 0.871)
	Ensemble	0.761	0.757	0.765	0.768	0.754	0.843 (0.783, 0.900)

*PPV* positive predictive value, *NPV* negative predictive value, *AUC* area under the curve, *CI* confidence interval

**Fig. 5:**
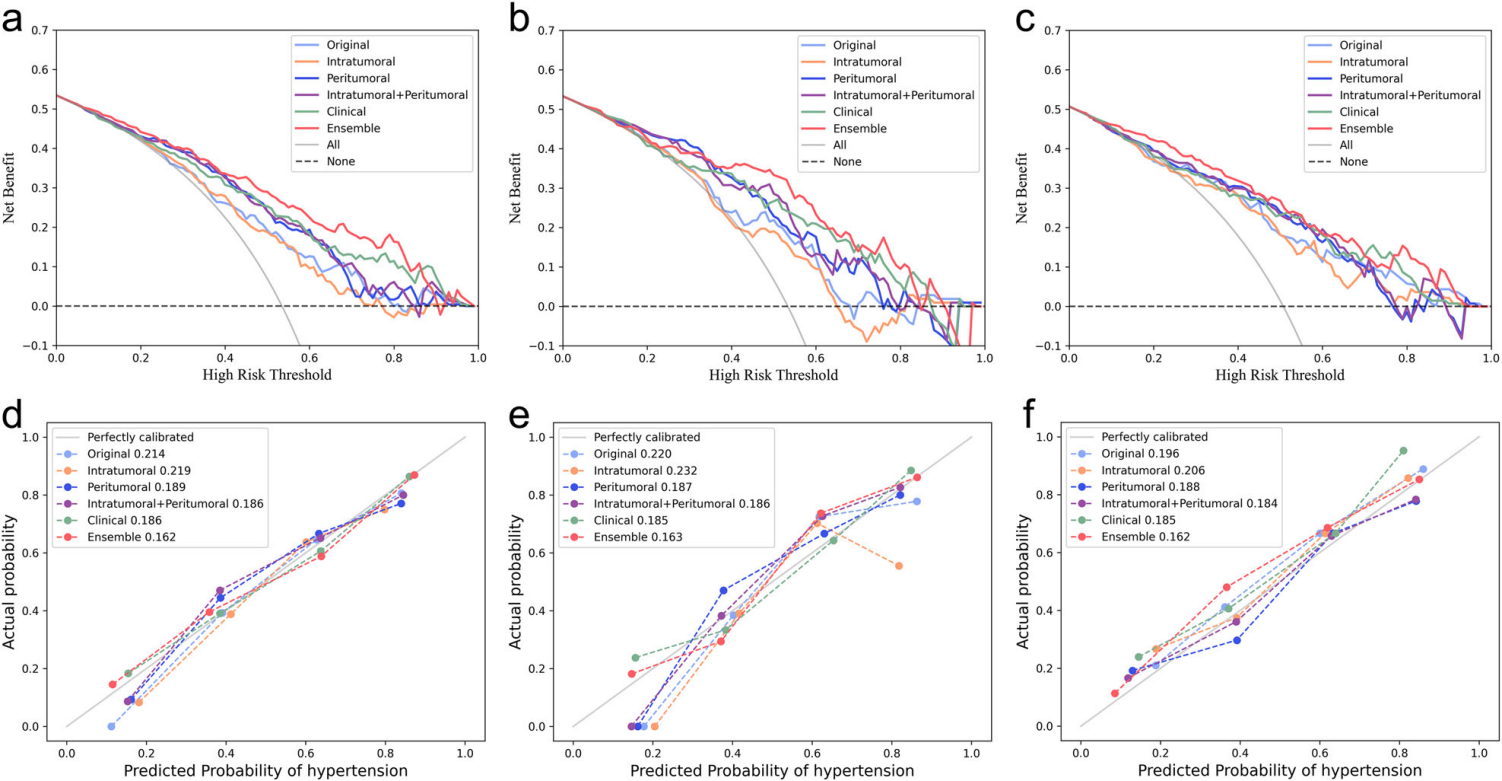
Decision curves of different models for predicting extrapelvic peritoneal metastasis in epithelial ovarian cancer on the training set (**a**), internal test set (**b**), and external test set (**c**). Calibration curves of the same models on the training set (**d**), internal test set (**e**), and external test set (**f**), while the numbers in the legend represent Brier scores corresponding to each model

**Fig. 6:**
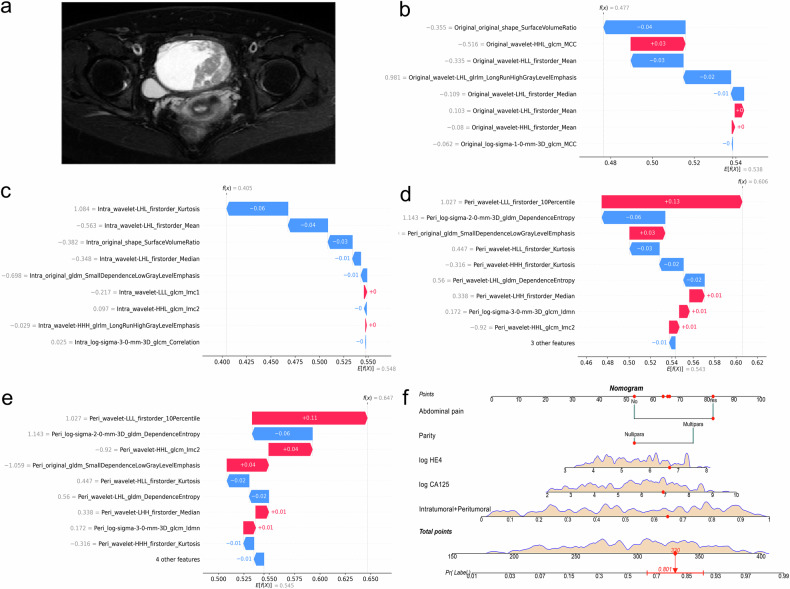
Interpretability analysis of models’ outputs for a 48-year-old patient with right high-grade serous carcinoma and with EPM. **a** Axial fat-suppressed T2-weighted image of the patient. **b**–**e** SHAP waterfall plots of different radiomics models for this patient. These plots reflect the impact of individual features on the models’ predictions. For each plot, the E[f(x)] is the baseline value, representing the average model output; each bar shows a feature’s contribution, with the length of the bar indicating the magnitude of the impact and the color indicating whether it increases (red) or decreases (blue) the prediction from the baseline; the f(x) refers to the model’s final prediction. **f** The prediction output of the ensemble model for this patient explained via a nomogram. “Intratumoral + peritumoral” denotes the output probability of the combined intratumoral and peritumoral model. From points scale at the top, the points contributed by each feature (red dots) are determined. Summing these points across all features gives the total points (red diamond), which can be mapped to the outcome scale at the bottom to find the predicted probability (red arrow) and its confidence interval (red line segment). EPM, extrapelvic peritoneal metastasis; SHAP, Shapley additive explanations

From the DCA, the ensemble model provided a greater net benefit than other models across various threshold probabilities (Fig. 5a–c). As indicated by the calibration curves and Brier scores, the ensemble model achieved superior accuracy relative to both the radiomics models and the clinical model by showing the highest concordance between predicted risk and observed probability (Fig. 5d–f). SHAP analysis results for radiomics models are shown in Fig. S6. One example of the model’s interpretability is illustrated in Fig. 6.

## Discussion

In the present study, we developed and validated multiple models to explore whether MRI-based peritumoral radiomics features can provide valuable information for predicting the EPM status of EOC patients preoperatively. The ensemble model, which incorporated the output of the combined intratumoral and peritumoral model with independent clinical predictors, exhibited optimal performance. The robustness and generalizability of the ensemble model were effectively demonstrated by its continued satisfactory performance across tenfold cross-validation, as well as on internal and external test sets.

The preoperative evaluation of EPM is a major challenge in the management of EOC. Previous studies using radiomics to predict the PM in EOC did not differentiate between intrapelvic PM and EPM [15, 16]. Notably, the presence of EPM indicates that the disease has progressed to a more advanced stage (at least FIGO III or T3 stage) than intrapelvic PM, crucial for clinicians to develop personalized treatment plans [5]. For example, patients with advanced EOC may benefit from NACT before debulking surgery, as it effectively reduces perioperative complications and mortality [23]; for early-stage EOC patients without EPM, fine needle aspiration biopsy should be avoided to prevent tumor rupture and the spillage of malignant cells into the peritoneal cavity [7]. The present study specifically focused on EPM, thereby providing results with direct clinical relevance. Additionally, the aforementioned studies were single-center with small sample sizes (*n* < 100) [15, 16]. In contrast, we included a multicenter sample of 488 patients, enhancing the result reliability. Furthermore, we employed tenfold cross-validation and both internal and external testing to evaluate our models, in line with the latest radiomics guidelines [24, 25]. These methods also improve the robustness and reproducibility of our findings.

In recent years, mounting evidence has highlighted the significant role of peritumoral microenvironment in the progression and metastasis of ovarian cancer [26–28]. However, previous radiomics-related studies on ovarian cancer mainly focused on tumor regions, ignoring the value of peritumoral areas [15, 16, 29]. Two studies showed a significant association between peritumoral radiomics features and the density of peripheral lymphocytes, suggesting that these features contain information related to the peritumoral microenvironment [30, 31]. In this study, the peritumoral model demonstrated superior AUC in predicting EPM status of EOC to the intratumoral model, especially in the internal test set (0.786 vs. 0.652, *p* = 0.007). This superiority may be attributed to peritumoral radiomics features effectively reflecting the dynamic changes in the tumor microenvironment, which is closely associated with metastasis in ovarian cancer [27]. Another possible explanation concerns the presence of cystic areas and frequent necrosis within EOC tumors, which may diminish the performance of the intratumoral model [3, 32]. The peritumoral model also achieved higher AUCs than the original model. This aligns with some previous studies, where identifying heterogeneity within peritumoral regions provided more specific insights into the tumor characteristics than whole tumor analyses [30, 33].

It is worth mentioning that there is no unified definition regarding the peritumoral area of ovarian tumors. One study on ovarian cancer utilized a 10 mm expansion to define the peritumoral ROI, potentially incorporating excessive non-tumoral tissue [17]. Another study reported that a 2 mm dilation provided optimal results in predicting the chemotherapy response of EOC [18]. However, a straightforward dilation approach may fail to capture the complex interactions between the tumor and the adjacent environment. Therefore, the peritumoral area in this study was defined by dilating the tumor boundary by 2 mm outward and shrinking it by 2 mm inward. This definition can precisely capture biologically significant areas while minimizing irrelevant noise, aligning with methodologies employed in studies on solid tumors and breast cancer [19, 20].

Recent studies showed that the combination of intratumoral and peritumoral information can enhance the capability of models for predicting metastasis in colorectal and cervical cancers, with AUCs of around 0.80 in the test sets [34, 35]. Consistent with these observations, we observed that this combination also improves performance in predicting EPM in EOC. We also observed that combining independent clinical predictors yielded the best diagnostic performance, with AUCs > 0.80 in both cross-validation and testing phases. These results are comparable to those of a previous MRI-based study, in which a model integrating deep learning with radiomics also achieved AUCs > 0.80 across different validation sets [29]. Notably, the ensemble model did not rely on deep learning, thus avoiding the need for substantial computational resources and potentially reducing both the technical barrier and costs.

Interpretability is crucial for the clinical application of models. In this study, we employed the SHAP method to perform interpretability analysis on radiomics models. This method provides the impact of different features on individual patient predictions, helping both clinicians and patients understand the model’s decision-making process. Additionally, we used a nomogram to visualize the ensemble model, allowing clinicians to intuitively interpret the model’s predictions. The use of these two interpretability analysis methods enhances the feasibility of our models in clinical practice.

Our study also suffers from some limitations. First, the retrospective nature of this study may lead to inherent biases. Thus, larger cohort sizes are necessary. Second, all models were developed based on FS-T2W images. Whether combining other sequences can yield additional valuable information needs further exploration. Third, only a 2 mm dilatation and shrinkage were selected as the peritumoral region in this study. Further studies are required to evaluate performance across different ranges of peritumoral areas. Finally, this study cannot determine the specific location, size, and extent of EPM.

In conclusion, peritumoral radiomics can offer valuable information regarding the EPM status in EOC patients. The ensemble model, which combines intratumoral and peritumoral radiomics with clinical characteristics, demonstrates optimal performance and has the potential to guide precision medicine for EOC patients.

## Supplementary information

ELECTRONIC SUPPLEMENTARY MATERIAL

## Data Availability

The datasets used and/or analyzed during the current study are available from the corresponding author upon reasonable request.

## Abbreviations

AUC: Area under the receiver operating characteristic curve
CA125: Carbohydrate antigen 125
CI: Confidence interval
DCA: Decision curve analysis
EOC: Epithelial ovarian cancer
EPM: Extrapelvic peritoneal metastasis
FIGO: International Federation of Gynecology and Obstetrics
FS-T2W: Fat-suppressed T2-weighted
HE4: Human epididymal protein 4
ICC: Intraclass correlation coefficient
MRI: Magnetic resonance imaging
NACT: Neoadjuvant chemotherapy
NPV: Negative predictive value
PM: Peritoneal metastasis
PPV: Positive predictive value
ROC: Receiver operating characteristic
ROI: Region of interest
SHAP: Shapley additive explanations
