# Supplementary material for: Intratumoral and peritumoral MRI-based radiomics for predicting extrapelvic peritoneal metastasis in epithelial ovarian cancer
Source: Insights Imaging. 2024 Nov 22;15:281. doi: 10.1186/s13244-024-01855-w (PMC11584833; doi:10.1186/s13244-024-01855-w)
Supplement: Supplementary file 1 — ELECTRONIC SUPPLEMENTARY MATERIAL [file 13244_2024_1855_MOESM1_ESM.pdf]

Intratumoral and Peritumoral MRI-based Radiomics for Predicting Extrapelvic Peritoneal Metastasis in Epithelial Ovarian Cancer

ELECTRONIC SUPPLEMENTARY MATERIAL

CONTENTS

Section 1 Center information and MRI parameters........................ 2

Section 2 Extrapelvic peritoneal metastasis 3

Section 3 Features extraction and selection 4

Section 4 Surgical findings 6

Tables 7

Figures 13

References.. 19

Section 1 Center information and MRI parameters

The multicenter study was conducted jointly by four centers:

Center I: the Affiliated Huaian No. 1 People's Hospital Hospital of Nanjing Medical University (3.0 T, Ingenia CX, Philips; 1.5/3.0 T, Aera/ Avanto/ Spectra/ Verio, SIEMENS)

Center II: the First Affiliated Hospital of Soochow University (1.5/3.0 T, Ingenia Ambition S/ Ingenia, Philips; 3.0 T, Signa HDxt, GE; 3.0 T, Skyra, SIEMENS).

Center III: Nantong Tumor Hospital (1.5/3.0 T, Espree/Verio, SIEMENS).

Center IV: the Affiliated Suzhou Hospital of Nanjing Medical University (1.5/3.0 T, Aera/Skyra, SIEMENS).

Scanners and imaging parameters of fat-suppressed T2-weighted (FS-T2W) from different centers are presented in **Table S1**.

Section 2 Extrapelvic peritoneal metastasis

Extrapelvic peritoneal metastasis (EPM) is defined as peritoneal metastasis above the pelvic brim and has a more advanced International Federation of Gynecology and Obstetrics (FIGO) stage than solely intrapelvic peritoneal metastasis. According to FIGO[1], EPM can include the following three conditions: 1) Endoscopic involvement of the extrapelvic peritoneum with or without positive retroperitoneal lymph nodes (FIGO IIIA/T3a2 stage); 2) Macroscopic presence of EPM, with a maximum diameter ≤ 2 cm, with or without retroperitoneal lymph node metastases (FIGO IIIB/T3b stage); 3) Macroscopic presence of EPM, with a maximum diameter > 2 cm, including tumor spread to the liver and splenic membranes but excluding organ parenchyma involvement, with or without retroperitoneal lymph node metastases (FIGO IIIC/T3c stage).

Section 3 Features extraction and selection

Features extraction

A total of 1130 radiomics features were extracted from each ROI. These included 14 shape features, 18 first-order features, 75 second-order features (including 24 gray level co-occurrence matrix, 16 gray level run length matrix, 16 gray level size zone matrix, 14 gray level dependence matrix, and 5 neighborhood gray-tone difference matrix), 279 features derived from the Laplacian-of-Gaussian filter, and 744 features derived from the wavelet filter. The extracted features conform to the Image Biomarker Standardization Initiative[2]. Detailed explanations and the formulas of these radiomics features are available at: https://pyradiomics.readthedocs.io/en/latest/features.html.

Features selection

The detailed feature selection steps were as follows: 1) The Mann-Whitney *U* test was employed to compare the differences in radiomics features between the EPM and non-EPM groups, after which features with a significance level of $P < 0.05$ were retained. 2) The random forest algorithm was utilized to determine the importance weight of each feature, and Spearman correlation analysis was conducted to calculate the correlation between pairs of features. For pairs with a correlation coefficient > 0.90, the feature with the lower importance weight was eliminated. 3) The least absolute shrinkage and

selection operator (LASSO) algorithm was applied to select features with non-zero coefficients, which helps reduce the multicollinearity.

To select features with high reproducibility, the training set underwent 100 random samplings, with 90% of the training data used for feature selection in each sampling. Features that appeared with a frequency of at least 50% after 100 iterations were utilized for the final radiomics model development. A similar frequency-based method of feature selection has also been applied in one study on distinguishing histological subtypes of EOC[3].

Section 4 Surgical findings

Among the 231 non-EPM patients, 191 showed no abnormalities of the peritoneum, while 40 had intrapelvic peritoneal metastasis. Specifically, metastasis involved the serosal layer of the rectum/sigmoid colon in 26 patients, the mesorectum in 5 patients, the serosal layer of the bladder/the peritoneal reflection of the bladder in 15 patients, and the pouch of Douglas in 12 patients. Ten of these patients had metastasis in two or more sites.

Among the 257 EPM patients, metastasis involved the omentum in 197 patients, the appendix in 82 patients, the unilateral/bilateral paracolic gutters in 65 patients, the serosal layer of colon in 54 patients, the serosal layer of small bowel in 22 patients, the mesocolon in 46 patients, the small bowel mesentery in 15 patients, the diaphragm in 51 patients, the liver capsule in 27 patients, and the spleen capsule in 4 patients. In this group, 161 patients had metastasis in two or more sites.

Tables

Table S1. Different Scanners and Imaging Parameters of FS-T2W.

Centers	Manufacturer	Type	Sequence	Magnetic Field (T)	TR (ms)	TE (ms)	Slice Thickness (mm)	Matrix Size
**Center I**	Philips	Ingenia CX	SE	3.0	4935	80	4~5	480×480
	SIEMENS	Aera	SE	1.5	4410~5140	86~87	5~6	640×640
	SIEMENS	Avanto	SE	1.5	1100~5420	96~120	5~8	384×512
	SIEMENS	Spectra	SE	3.0	4500	96	4.5~5.5	640×640
	SIEMENS	Verio	SE	3.0	1700~5000	97~98	5~7	320×320/240×320
**Center II**	Philips	Ingenia Ambition S	SE	1.5	3333~4843	85~90	5~6.5	384×384/512×512
	Philips	Ingenia	SE	3.0	3385~4776	94~12	5~6	576×576/640×640
	GE	Signa HDxt	SE	3.0	4120	104	6	512×512
	SIEMENS	Skyra	SE	3.0	5000	77	5~6	640×640
**Center III**	SIEMENS	Espree	SE	1.5	3500~5630	63~74	4~5.5	180×256
	SIEMENS	Verio	SE	3.0	3810	64	4~5	3220×320
**Center IV**	SIEMENS	Skyra	SE	3.0	3020	101	6~9	320×320
	SIEMENS	Aera	SE	1.5	5250	85	6	320×320

FS-T2W, fat-suppressed T2-weighted; TR, repetition time; TE, echo time.

Table S2. Demographic and Clinical Characteristics of Patients in Training Set.

	Non-EPM (n = 114)	EPM (n = 131)	*P* value
**Age**			0.438
< 55	57 (50.0%)	59 (45.0%)	
≥ 55	57 (50.0%)	72 (55.0%)	
**Menopausal status**			0.412
Premenopausal	20 (17.5%)	18 (13.7%)	
Postmenopausal	94 (82.5%)	113 (86.3%)	
**Parity**			0.024*
Nullipara	35 (30.7%)	24 (18.3%)	
Multipara	79 (69.3%)	107 (81.7%)	
**Abdominal pain**			< 0.001*
No	71 (62.3%)	46 (35.1%)	
Yes	43 (37.7%)	85 (64.9%)	
**Abdominal distention**			0.013*
No	72 (63.2%)	62 (47.3%)	
Yes	42 (36.8%)	69 (52.7%)	
**FIGO stage**			< 0.001*
I/II	102 (89.5%)	0 (0.0%)	
III/IV	12 (10.5%)	131 (100.0%)	
**Type**			< 0.001*
I	60 (52.6%)	16 (12.2%)	
II	54 (47.4%)	115 (87.8%)	
**log CA125 (u/mL)**	4.94±1.55	6.34±1.53	< 0.001*
**log HE4 (pmol/L)**	4.95±0.93	5.88±1.02	< 0.001*
**Pathology subtype**			< 0.001*
HGSC	54 (47.4%)	115 (87.8%)	
LGSC	9 (7.9%)	4 (3.1%)	
Clear cell	14 (12.3%)	7 (5.3%)	
Endometrioid	21 (18.4%)	1 (0.8%)	
Mucinous	15 (13.2%)	3 (2.3%)	
Brenner	1 (0.9%)	1 (0.8%)	

Categorical variables are presented as numbers (%) and continuous variables are presented as mean ± standard deviation. EPM, extrapelvic peritoneal metastasis; FIGO, International Federation of Gynecology and Obstetrics; CA125, carbohydrate antigen 125; HE4, human epididymis protein 4; HGSC, high-grade serous carcinoma; LGSC, low-grade serous carcinoma.

*, $p < 0.05$.

Table S3. Demographic and Clinical Characteristics of Patients in Internal Test Set.

	**Non-EPM (n = 49)**	**EPM (n = 56)**	***P* value**
**Age**			0.100
< 55	28 (57.1%)	23 (41.1%)	
≥ 55	21 (42.9%)	33 (58.9%)	
**Menopausal status**			0.576
Premenopausal	8 (16.3%)	7 (12.5%)	
Postmenopausal	41 (83.7%)	49 (87.5%)	
**Parity**			0.406
Nullipara	11 (22.4%)	9 (16.1%)	
Multipara	38 (77.6%)	47 (83.9%)	
**Abdominal pain**			0.210
No	27 (55.1%)	24 (42.9%)	
Yes	22 (44.9%)	32 (57.1%)	
**Abdominal distention**			< 0.001*
No	35 (71.4%)	21 (37.5%)	
Yes	14 (28.6%)	35 (62.5%)	
**FIGO stage**			< 0.001*
I/II	42 (85.7%)	0 (0.0%)	
III/IV	7 (14.3%)	56 (100.0%)	
**Type**			< 0.001*
I	27 (55.1%)	6 (10.7%)	
II	22 (44.9%)	50 (89.3%)	
**log CA125 (u/mL)**	4.88±1.53	6.29±1.20	< 0.001*
**log HE4 (pmol/L)**	4.73±0.82	5.84±0.95	< 0.001*
**Pathology subtype**			< 0.001*
HGSC	22 (44.9%)	50 (89.3%)	
LGSC	3 (6.1%)	3 (5.4%)	
Clear cell	10 (20.4%)	1 (1.8%)	
Endometrioid	6 (12.2%)	0 (0.0%)	
Mucinous	8 (16.3%)	1 (1.8%)	
Seromucinous	0 (0.0%)	1 (1.8%)	

Categorical variables are presented as numbers (%) and continuous variables are presented as mean ± standard deviation. EPM, extrapelvic peritoneal metastasis; FIGO, International Federation of Gynecology and Obstetrics; CA125, carbohydrate antigen 125; HE4, human epididymis protein 4; HGSC, high-grade serous carcinoma; LGSC, low-grade serous carcinoma.

*, $p < 0.05$.

Table S4. Demographic and Clinical Characteristics of Patients in External Test Set.

	Non-EPM (n = 68)	EPM (n = 70)	*P* value
**Age**			0.062
< 55	38 (55.9%)	28 (40.0%)	
≥ 55	30 (44.1%)	42 (60.0%)	
**Menopausal status**			0.379
Premenopausal	9 (13.2%)	6 (8.6%)	
Postmenopausal	59 (86.8%)	64 (91.4%)	
**Parity**			0.390
Nullipara	22 (32.4%)	18 (25.7%)	
Multipara	46 (67.6%)	52 (74.3%)	
**Abdominal pain**			0.684
No	45 (66.2%)	44 (62.9%)	
Yes	23 (33.8%)	26 (37.1%)	
**Abdominal distention**			0.067
No	49 (72.1%)	40 (57.1%)	
Yes	19 (27.9%)	30 (42.9%)	
**FIGO stage**			< 0.001*
I/II	61 (89.7%)	0 (0.0%)	
III/IV	7 (10.3%)	70 (100.0%)	
**Type**			< 0.001*
I	43 (63.2%)	13 (18.6%)	
II	25 (36.8%)	57 (81.4%)	
**log CA125 (u/mL)**	4.37±1.44	5.98±1.19	< 0.001*
**log HE4 (pmol/L)**	4.58±0.71	5.84±0.93	< 0.001*
**Pathology subtype**			< 0.001*
HGSC	25 (36.8%)	54 (77.1%)	
LGSC	5 (7.4%)	3 (4.3%)	
Clear cell	20 (29.4%)	1 (1.4%)	
Endometrioid	8 (11.8%)	6 (8.6%)	
Mucinous	10 (14.7%)	3 (4.3%)	
Carcinosarcoma#	0 (0.0%)	2 (2.9%)	
Brenner	0 (0.0%)	1 (1.4%)	

Categorical variables are presented as numbers (%) and continuous variables are presented as mean ± standard deviation. EPM, extrapelvic peritoneal metastasis; FIGO, International Federation of Gynecology and Obstetrics; CA125, carbohydrate antigen 125; HE4, human epididymis protein 4; HGSC, high-grade serous carcinoma; LGSC, low-grade serous carcinoma.

*, $p < 0.05$.

#, Carcinosarcoma is defined as epithelial in origin according to the latest World Health Organization classification criteria.

Table S5. Results of Multivariate Logistic Regression Analysis.

Variables	Odds ratios	95% CI	*P* value
log CA125	1.47	(1.16, 1.85)	0.001*
log HE4	1.68	(1.17, 2.40)	0.005*
Parity	2.15	(1.06, 4.35)	0.033*
Abdominal pain	3.10	(1.70, 5.68)	< 0.001*
Abdominal distention	1.33	(0.73, 2.42)	0.356

CI, confidence interval; CA125, carbohydrate antigen 125; HE4, human epididymis protein 4.

*, $p < 0.05$.

Table S6. Delong Tests between AUCs of Different Models.

Models	Internal test set		External test set	
	Z score	*P* value	*Z* score	*P* value
**Original** vs. **Intratumoral**	1.285	0.199	1.277	0.202
**Original** vs. **Peritumoral**	-2.242	0.025*	-0.162	0.871
**Peritumoral** vs. **Intratumoral**	2.682	0.007*	1.244	0.213
**Intratumoral + Peritumoral** vs. **Intratumoral**	3.070	0.002*	1.513	0.130
**Intratumoral + Peritumoral** vs. **Peritumoral**	0.138	0.890	0.584	0.559
**Intratumoral + Peritumoral** vs. **Original**	2.686	0.007*	0.321	0.748
**Intratumoral + Peritumoral** vs. **Clinical**	-0.115	0.909	-0.249	0.803
**Clinical** vs. **Intratumoral**	2.434	0.015*	1.117	0.264
**Clinical** vs. **Peritumoral**	0.149	0.882	0.334	0.738
**Clinical** vs. **Original**	1.809	0.070	0.475	0.635
**Ensemble** vs. **Intratumoral**	3.565	< 0.001*	2.669	0.008*
**Ensemble** vs. **Peritumoral**	1.248	0.212	1.984	0.047*
**Ensemble** vs. **Original**	3.196	0.001*	1.896	0.058
**Ensemble** vs. **Intratumoral + Peritumoral**	1.349	0.177	1.918	0.055
**Ensemble** vs. **Clinical**	1.635	0.102	1.466	0.143

AUC, area under the curve.

*, $p < 0.05$.

Figures

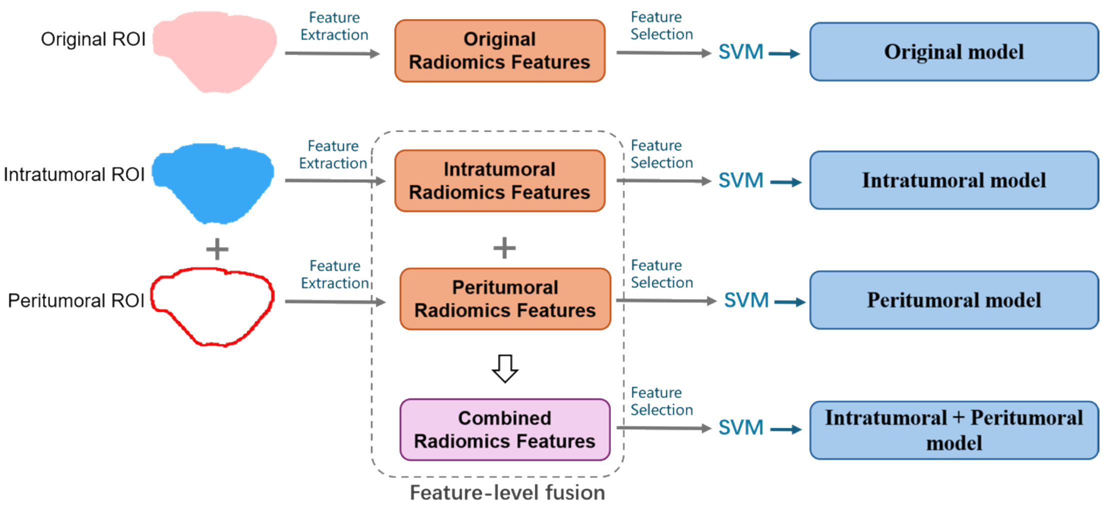

Figure S1. Process of constructing different radiomics models. The feature-level fusion method is indicated within the dashed box. ROI, region of interest; SVM, support vector machine.

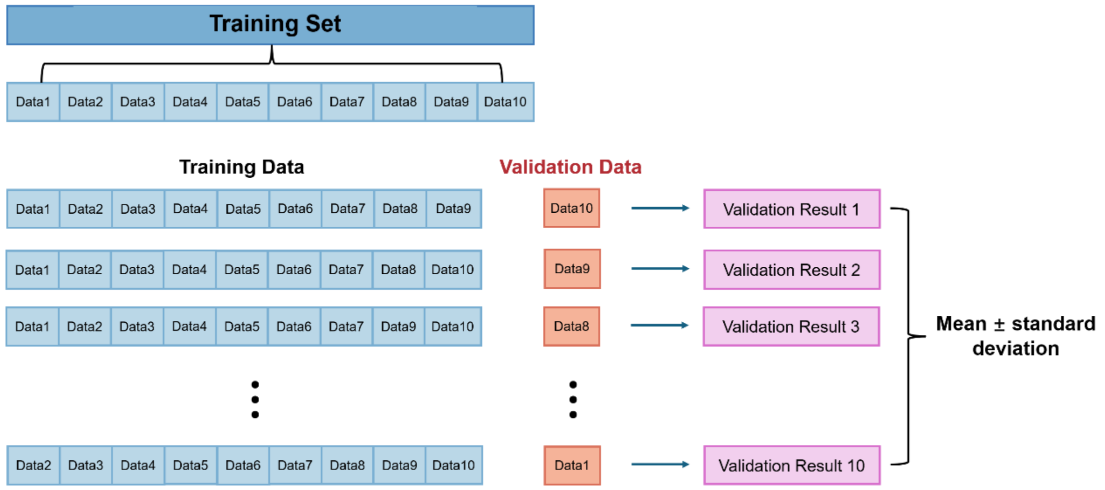

Figure S2. The schematic diagram of 10-fold cross-validation. In each iteration of cross-validation, the training set is randomly divided into 10 approximately equal-sized subsets. Nine of these subsets are used as training data, while the remaining subset is used as validation data. The final results across 10-fold cross-validation are presented as the mean ± standard deviation.

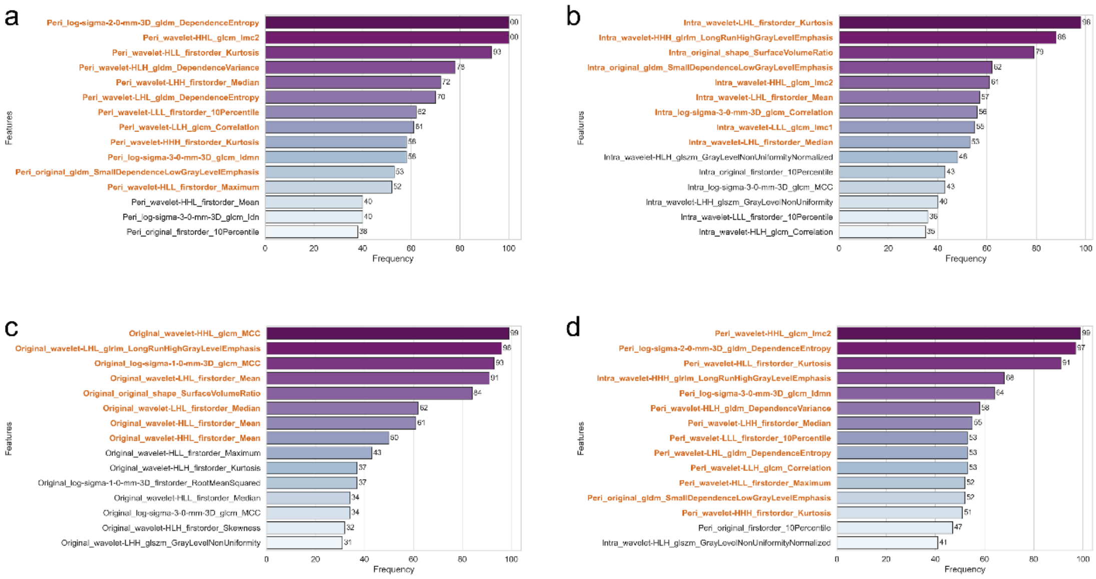

Figure S3. Bar charts of the top 15 radiomics features from the peritumoral ROI (a), intratumoral ROI (b), original ROI (c), and combined intratumoral and peritumoral features (d) after feature selection. Features marked in orange appear with a frequency of at least 50% after the feature selection process, which involved 100 iterations. ROI, region of interest.

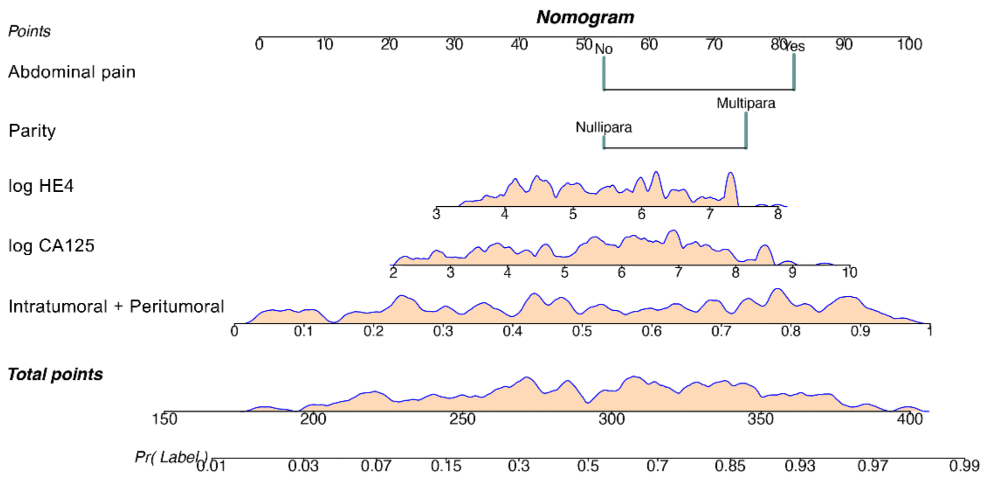

Figure S4. The nomogram for the ensemble model. The “Intratumoral + Peritumoral” denotes the output probability of the combined intratumoral and peritumoral model.

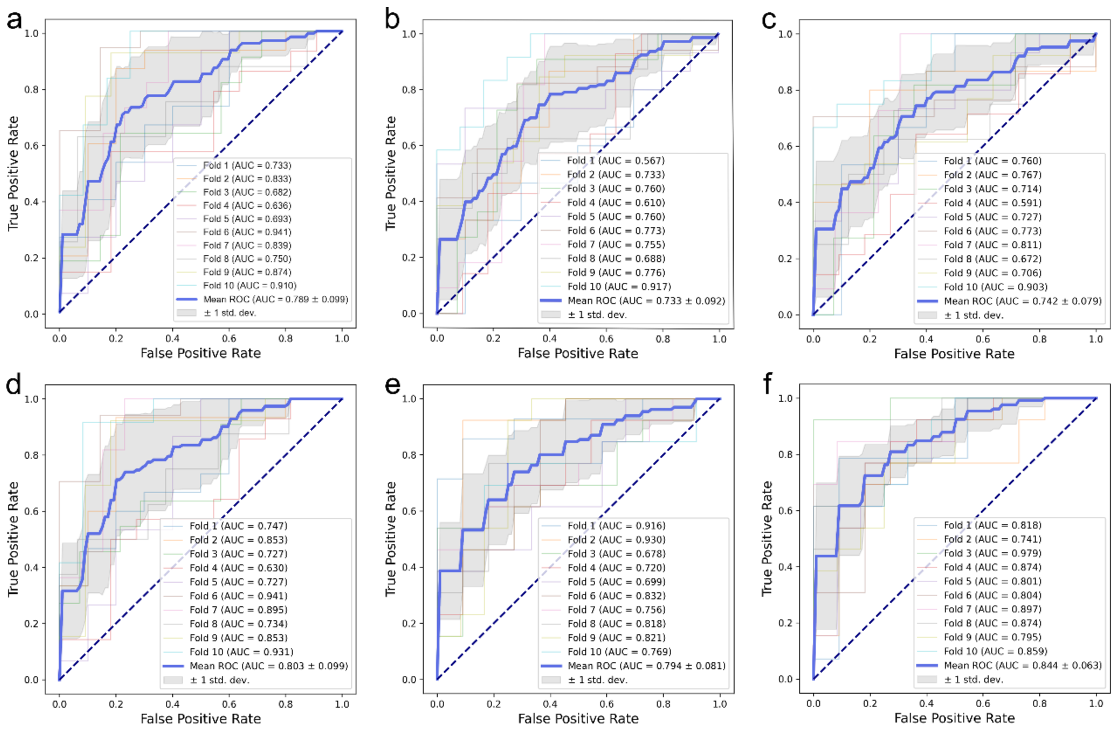

Figure S5. The 10-fold cross-validation ROC curves of peritumoral model (a), intratumoral model (b), original model (c), combined intratumoral and peritumoral model (d), clinical model (e) and ensemble model (f) for predicting extrapelvic peritoneal metastasis in epithelial ovarian cancer. For each plot, each light-colored line represents one curve of the 10 folds (folds 1 to 10); the bold blue curve represents mean ROC curve; the gray area represents the standard deviation. ROC, receiver operating characteristic; AUC, area under the curve.

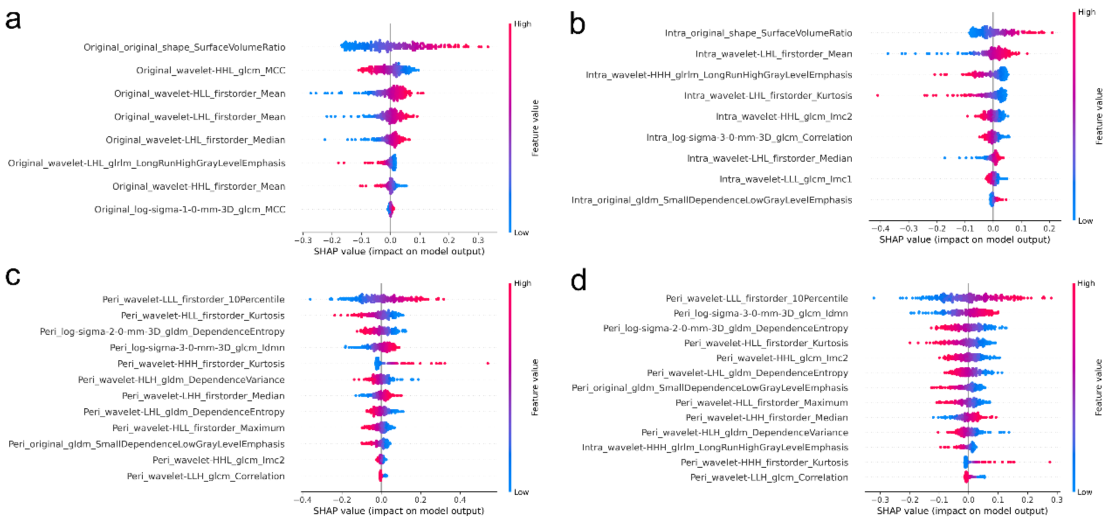

Figure S6. SHAP summary plots of the original (a), intratumoral (b), peritumoral (c), and combined intratumoral and peritumoral models (d). These plots interpret the output of models by illustrating the impact of each feature on the models' predictions. For each plot, the X-axis represents SHAP values, indicating the contribution of features to the prediction, with positive values increasing the prediction and negative values decreasing it; the Y-axis lists features, sorted by their average importance; each point represents an observation, with the color indicating the feature value (from blue for low values to red for high values).

References

1 Berek JS, Renz M, Kehoe S, Kumar L, Friedlander M (2021) Cancer of the ovary, fallopian tube, and peritoneum: 2021 update. International Journal of Gynecology & Obstetrics DOI: 10.1002/ijgo.13878

2 Zwanenburg A, Vallières M, Abdalah MA et al (2020) The Image Biomarker Standardization Initiative: Standardized Quantitative Radiomics for High-Throughput Image-based Phenotyping. Radiology DOI: 10.1148/radiol.2020191145

3 Wang M, Perucho JAU, Hu Y et al (2022) Computed Tomographic Radiomics in Differentiating Histologic Subtypes of Epithelial Ovarian Carcinoma. JAMA Network Open DOI: 10.1001/jamanetworkopen.2022.45141
